# A Review of Research Progress in Rice Anther Culture

**DOI:** 10.3390/cimb48010018

**Published:** 2025-12-24

**Authors:** Zhizun Feng, Huangwei Chu, Liming Cao, Ruiyun Wang, Anpeng Zhang

**Affiliations:** 1 College of Agronomy, Shanxi Agricultural University, Taigu 030801, China; 2 Key Laboratory of Germplasm Innovation and Genetic Improvement of Grain and Oil Crops (Co-Construction by Ministry and Province), Ministry of Agriculture and Rural Affairs, Crop Breeding and Cultivation Research Institute, Shanghai Academy of Agricultural Sciences, Shanghai 201403, China; 3 Center for Agricultural Genetic Resources Research, Shanxi Agricultural University/Key Laboratory of Crop Gene Resources and Germplasm Enhancement on Loess Plateau, Ministry of Agriculture and Rural Affairs/Shanxi Key Laboratory of Genetic Resources and Genetic Improvement of Minor Crops, Taiyuan 030031, China

**Keywords:** anther culture, haploid induction, doubled haploid, rice breeding, in vitro culture

## Abstract

Conventional rice breeding predominantly relies on hybridization techniques, with hybrid progenies typically requiring 8 to 10 generations of selfing to achieve genetically stable homozygous lines. In contrast, haploid breeding enables the derivation of stable doubled haploid (DH) lines from hybrid progeny in just one generation, substantially shortening the breeding cycle. Haploid breeding comprises two core steps: haploid induction and chromosome doubling, with efficient haploid induction being pivotal to the success of this technology. Currently, anther culture, due to its relatively mature and stable protocol, has become the primary method for obtaining haploids in rice haploid breeding. This review systematically summarizes the research progress in rice anther culture, focusing on the fundamental steps and applications of haploid breeding, the developmental history of anther culture, factors influencing anther culture efficiency and their underlying genetic mechanisms, current challenges and potential countermeasures, and future prospects for rice anther culture technology.

## 1. Introduction

**Figure 1:**
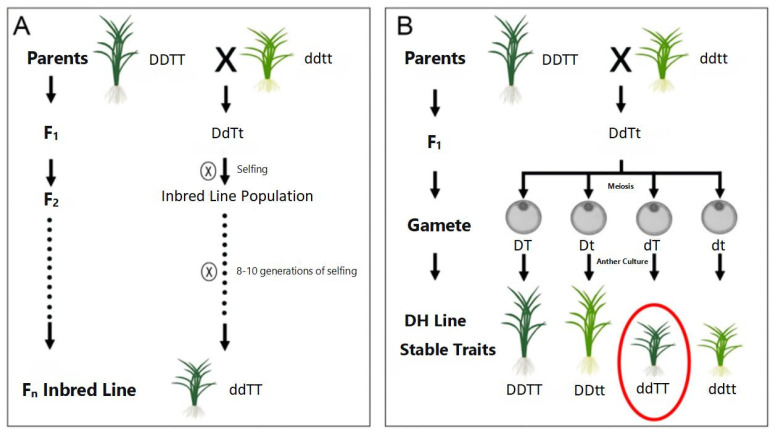
Comparison of breeding procedures between hybrid breeding (**A**) and haploid breeding (**B**). Note: ⊗: Selfing; X: Crossing; A dashed (or dotted) line with an arrow represents propagation over multiple generations; A solid arrow line represents propagation for a single generation; A red circle indicates the breeding objective to be obtained.

Hybrid breeding stands as one of the most widely utilized methods in crop improvement. This approach typically involves crossing or backcrossing parental lines with distinct traits, followed by multiple generations of selfing combined with rigorous selection and evaluation, ultimately yielding pure lines that incorporate desirable characteristics from both parents and possess genetic stability [1]. However, conventional hybrid breeding is hampered by labor-intensive procedures, extended breeding cycles, and low efficiency in selecting elite cultivars. The development of haploid breeding technology has introduced a transformative breakthrough; its remarkable efficiency has earned it the moniker of the “high-speed rail of crop breeding”. The core protocol involves inducing haploid (containing half the somatic chromosome number) plants from hybrid or backcross progeny, followed by chromosome doubling to generate doubled haploid (DH) lines. In contrast to the 8–10 generations required by traditional hybrid breeding to achieve genetically stable pure lines, haploid breeding enables the production of stable DH lines within a single generation, drastically shortening the breeding cycle and significantly enhancing breeding efficiency (Figure 1). The completely homozygous genome of DH lines allows for more precise phenotypic selection, proving particularly advantageous for the efficient screening of quantitative traits [2]. To date, haploid breeding techniques have been successfully applied in over 200 plant species [3], establishing them as a rapid, cost-effective, and highly efficient strategy in modern crop breeding.

Rice, as the most crucial food crop in China, has consistently attracted significant research attention in the field of haploid breeding. Currently, haploid induction in rice is primarily achieved through two methodological approaches: anther culture and inducer-mediated induction. Among these, anther culture remains the predominant technique for generating haploids in practical rice breeding programs. The standard protocol involves the aseptic inoculation of anthers at the uninucleate microspore stage onto an induction medium. This process enables the haploid microspores—products of meiosis—to undergo dedifferentiation and form callus. Subsequently, the callus is induced to redifferentiate into haploid plantlets, which ultimately develop into fertile double haploid (DH) plants through natural or artificially facilitated chromosome doubling. The integration of in vitro culture techniques is indispensable to this entire process. It provides a sterile, controlled environment that allows for the precise manipulation of factors (nutrients, hormones, temperature, light) crucial for inducing and sustaining microspore development outside the living plant. This capability not only enables the rapid production of DH lines in a matter of months, bypassing years of selfing, but also facilitates studies on fundamental aspects of cell totipotency, embryogenesis, and genetic regulation under standardized laboratory conditions [4,5].

This review was conducted following a systematic approach to identify relevant literature. A comprehensive search was performed using the core academic databases Web of Science, Scopus, PubMed, and the China National Knowledge Infrastructure (CNKI). The search strategy employed key Boolean combinations of terms including: “rice” OR “*Oryza sativa*”, “anther culture” OR “microspore culture”, “haploid” OR “doubled haploid”, and “breeding”. The publication timeline was focused from the foundational works in the 1960s up to the latest available studies in 2025. Articles were initially screened based on title and abstract for relevance to rice anther culture techniques, efficiency factors, genetic studies, and breeding applications. Selected full-text papers were then evaluated and synthesized to construct this narrative review.

Building upon a foundational overview of the haploid breeding technology system, this review systematically examines the historical development of rice anther culture. It further analyzes factors influencing anther culture efficiency at both physiological and genetic levels, proposes strategic countermeasures to existing challenges, and outlines future research directions. This work aims to provide a theoretical framework and reference for subsequent studies in this field.

## 2. Fundamental Steps and Applications of Haploid Breeding

### 2.1. Haploid Induction

Haploid plant induction can be achieved through two principal pathways: in vivo and in vitro.

In vivo pathways primarily encompass inducer-mediated induction, pollen induction, wide hybridization, and centromere-mediated genome elimination. Inducer-mediated induction utilizes inducer lines carrying specific genes, which are crossed with recipient plants to promote haploid formation. Recent advances in functional genomics and gene editing have enabled the successful development of haploid inducer lines in rice [6]. However, a significant challenge for practical application remains enhancing the efficiency of cross-pollination, as rice is a self-pollinating species. Pollen induction involves treating pollen with physical or chemical agents prior to pollination, causing damage to its genetic material. This treatment induces the formation of haploid plants from the egg cell or other embryo sac cells within the recipient plant. This method has been documented in important crops such as wheat, maize, and tobacco (*Nicotiana tabacum* L.) [7]. Wide hybridization, involving crosses between different species or genera, exploits chromosomal instability to induce haploid formation in the embryo sac of the recipient plant. A notable example is the generation of wheat haploids following hybridization with maize, resulting from the elimination of maize chromosomes [8]. Centromere-mediated genome elimination relies on functional inactivation or aberrant division of chromosome centromeres, leading to chromosome loss during mitosis or meiosis and subsequent haploid formation. For instance, manipulation of the *CENH3* gene, which encodes a centromere-specific histone H3 variant, can cause selective elimination of non-parental chromosomes in hybrid embryos, thereby producing haploid plants [9].

In vitro pathways primarily include anther culture, microspore culture, and gynogenesis. Among these, anther culture is the most frequently employed method for haploid production. This technique involves the in vitro culture of anthers at the late uninucleate stage (also referred to as the late microspore stage) on induction medium. Microspores are induced to undergo dedifferentiation, forming callus. This callus is then transferred to a differentiation medium, where it regenerates into haploid plants. China pioneered the application of rice anther culture in breeding as early as the 1970s [10] and has since successfully developed numerous important rice varieties using this method [11]. To date, anther culture has been successfully established in various crops, including rice (*Oryza sativa* L.), maize (*Zea mays* L.), and wheat (*Triticum aestivum* L.) [12]. The principle of microspore culture is similar to that of anther culture; the key distinction lies in the isolation of microspores from the anthers. These isolated microspores are then cultured on induction medium to form callus, which subsequently differentiates into haploid plants [13]. This technique is widely used in breeding and genetic research of important crops like wheat [14]. Gynogenesis involves the in vitro culture of embryo sacs or ovules, inducing their development into haploid plants, a process analogous to parthenogenesis. Due to the limited number and difficulty in isolating female gametes, this method sees relatively little use in practical breeding. It currently finds some application in species such as sugar beet (*Beta vulgaris* L.) and onion (*Allium cepa* L.) [15].

In summary, despite the rapid development of emerging in vivo induction techniques, anther culture remains the principal technological approach in rice haploid breeding, owing to its well-established and robust protocol.

### 2.2. Chromosome Doubling

Haploid plants are inherently sterile and require chromosome doubling to produce fertile double haploid (DH) plants for subsequent genetic analysis and stable line breeding. Chromosome doubling techniques primarily comprise three approaches: chemical induction, physical induction, and spontaneous doubling.

Chemical induction, the most prevalent method, achieves chromosome doubling by inhibiting spindle formation during cell division, thereby preventing chromosome segregation and retaining complete chromosome sets in daughter cells. Common chemical inducers include colchicine, oryzalin, and cytochalasin B. Colchicine, a microtubule polymerization inhibitor, disrupts spindle assembly to block chromosome separation during mitosis. It is typically applied through root-tip soaking, seedling spraying, or in vitro tissue treatment. Oryzalin, exhibiting similar spindle-disrupting effects via microtubule inhibition but with lower toxicity, has gained increasing application in plant chromosome doubling. Cytochalasin B is also employed for specific species. Wan et al. [16] demonstrated that low-concentration colchicine treatment significantly enhanced diploid plant production in maize anther culture, while analogous studies have been reported in rice [17].

Physical induction modifies chromosome division through cellular or tissue manipulation, utilizing techniques such as temperature stress (low/high), mechanical injury, radiation exposure (γ, β, α rays, UV), and high-ion concentration treatment (e.g., elevated Ca^2+^). For instance, prolonged cold treatment substantially increases spontaneous doubling rates in spring wheat [18], and heat treatment has been successfully applied for chromosome doubling in pepper (*Capsicum annuum* L.), significantly improving haploid breeding efficiency [19].

Spontaneous doubling occurs without external intervention during developmental processes, resulting from spindle abnormalities, chromosome missegregation, or cell cycle dysregulation in either somatic mitosis or meiosis. The spontaneous doubling rate varies across species and culture methods: 20–30% in anther-derived and 30–40% in microspore-derived wheat haploid plants [3], whereas maize pollen-induced haploid plants rarely undergo spontaneous doubling and necessitate chemical induction [3]. Rice anther culture yields haploid plants with a 30–40% spontaneous doubling rate [20].

### 2.3. Applications of DH Populations in Genetics and Breeding

DH populations exhibit extensive applications in both genetic research and plant breeding. In genetic studies, the completely homozygous nature of DH populations makes them invaluable material for constructing genetic linkage maps, conducting quantitative trait locus (QTL) analysis, and performing gene mapping. To date, genetic linkage maps developed from DH populations have been widely utilized in genetic research of major crops such as rice [21], maize [22], and wheat [23], providing crucial support for QTL analysis of complex traits and fine mapping of genes. He et al. [24] employed a DH population derived from an indica/japonica cross and detected five QTLs for callus induction rate on chromosomes 6, 7, 8, 10, and 12, two QTLs for green plant differentiation rate on chromosomes 1 and 9, and one major QTL for albino plant differentiation rate on chromosome 9 in rice. Zhang et al. [25] used a DH population derived from a cross between japonica variety CJ06 and indica variety TN1, and through QTL analysis, identified 13 QTLs associated with grain shape, amylose content, and Rapid Viscosity Analysis (RVA) profile characteristics, providing a foundation for molecular breeding of rice quality. These genomic regions present potential targets for future genetic improvement, fine mapping, and marker-assisted selection. Yang et al. [26] used a spring wheat DH population comprising 174 lines to detect and map QTLs associated with Fusarium head blight (FHB) resistance. Single-locus QTL analysis identified seven QTLs for Type I resistance (resistance to initial infection), four QTLs for Type II resistance (resistance to within-spread disease), and six QTLs for kernel infection resistance. Furthermore, two-locus QTL analysis detected eight main-effect QTLs and four additive × additive epistatic interaction pairs, and novel FHB resistance genes were identified on chromosomes 1DL, 4AL, and 4DL for the first time. These findings provide important theoretical foundations for elucidating the genetic mechanisms of FHB resistance and guiding resistance breeding.

In breeding research, DH technology significantly accelerates crop breeding cycles and facilitates the establishment of rapid breeding systems. The ability of DH populations to achieve complete homozygosity within a single generation drastically shortens the breeding timeline and enhances breeding efficiency compared to traditional successive selfing methods [27]. Furthermore, DH technology finds valuable applications in hybrid breeding, resistance breeding, and the improvement of desirable agronomic traits. It has been successfully implemented in the breeding programs of various crops. In rice breeding, substantial progress has been made in developing efficient haploid inducer lines. Researchers have successfully created rice haploid inducer lines with an induction efficiency of 12.4%, which have been applied to the breeding of two-line sterile lines, enabling the large-scale application of DH technology in rice improvement [6]. In maize breeding, the use of DH populations allows for the rapid generation of homozygous lines, greatly facilitating the dissection of complex trait inheritance and the development of hybrid cultivars [28]. In wheat breeding, DH technology effectively shortens the selection cycle for elite varieties with enhanced disease resistance and yield potential by rapidly generating homozygous germplasm resources [29].

The technical pathway of haploid breeding fundamentally comprises two core components: successful haploid induction and subsequent chromosome doubling. Among these, haploid induction is the pivotal step. The resulting doubled haploid (DH) populations, characterized by their homozygous genetic background and stable trait expression, have become indispensable materials for modern genetic research and breeding design. Within the current technological framework, anther culture remains the most mature and reliable method to achieve this objective. Therefore, systematically elucidating the genetic mechanisms governing anther culture efficiency and overcoming its technical bottlenecks represent central challenges for advancing haploid breeding in rice.

## 3. Historical Development of Rice Anther Culture

**Figure 2:**
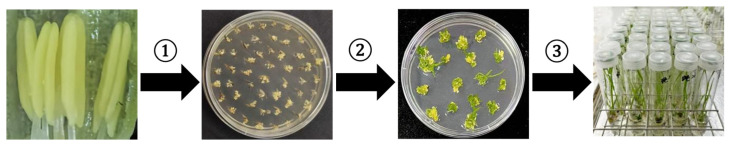
Procedure of anther culture in rice. Note: ①: Callus induction; ②: Shoot differentiation; ③: Rooting culture.

Rice anther culture technology serves as a pivotal method for generating haploid plants by inducing microspore development. The core procedural steps encompass: (1) Selection of anthers at the late uninucleate stage, followed by aseptic inoculation onto induction medium to exploit microspore totipotency for callus formation through dedifferentiation; (2) Transfer of callus (upon reaching approximately 2 mm in size) to differentiation medium to promote shoot regeneration; and (3) Transplantation of differentiated plantlets to rooting medium to facilitate the regeneration of complete plants (Figure 2).

In 1921, Bergner first reported naturally occurring haploid plants in jimsonweed (*Datura stramonium* L.) [30]. These plants exhibited characteristics such as reduced plant height, smaller leaves, and delayed development. Cytological analysis revealed that their chromosome number (*n* = 12) was half that of normal diploids (2*n* = 24). This discovery laid the foundation for plant haploid biology and subsequently promoted the development of haploid breeding technologies. In 1964, Guha and Maheshwari achieved the first successful production of haploid plants using anther culture in jimsonweed [31]. They demonstrated that culturing immature anthers on a suitable medium could induce microspores to develop into embryoids, ultimately generating complete haploid plants. This finding confirmed that, under specific conditions, microspores can switch from normal gametophytic development to embryogenic development, thereby establishing the fundamental basis for anther culture technology and providing critical support for subsequent haploid breeding and cytogenetic research.

Rice anther culture technology was first successfully reported in 1968 [32]. China initiated research on rice anther culture technology and its application in rice breeding in 1970. In 1975, the Crop Research Institute of Heilongjiang Academy of Agricultural Sciences pioneered the use of anther culture breeding to develop the japonica rice variety ‘Danfeng 1’, marking the initiation of anther culture technology in Chinese rice breeding [33]. Subsequently, Chinese rice breeders successfully cultivated numerous high-quality rice varieties using anther culture, including the Zhonghua series (Zhonghua 8 to Zhonghua 14) developed by the Chinese Academy of Agricultural Sciences, the Longjing series bred by the Heilongjiang Academy of Agricultural Sciences, and the Huayu series (Huayu 1, Huayu 2, Huayu 3, Huayu 13, Huayu 560, etc.) developed by the Tianjin Crop Research Institute. According to statistics, the cumulative planting area of rice varieties developed through anther culture reached 910,000 hectares between 1975 and 1995 (a 21-year period). In contrast, from 1996 to 1998 (a 3-year period), the planting area reached 950,000 hectares, surpassing the total of the previous 21 years. By 1998, over 40 varieties had been approved [11]. Through nearly half a century of application in China, anther culture technology has achieved notable success in rice breeding. However, its efficiency remains highly genotype-dependent. The particular difficulty in culturing anthers of indica varieties has limited the broader application of this technique in breeding programs. Currently, only a limited number of breeding institutions continue to employ this technology (Table 1).

## 4. Factors Influencing the Efficiency of Rice Anther Culture

The efficiency of rice anther culture is influenced by multiple factors, including genotype, sampling stage and position, pretreatment, growth environment, medium composition, and culture conditions. Among these, genotype is the most critical determinant.

Significant variation in anther culturability exists among different rice varieties, primarily attributable to their distinct genetic backgrounds. A marked difference is observed between indica and japonica subspecies, with japonica varieties generally exhibiting higher anther culture efficiency than their indica counterparts [34]. This disparity is likely governed by fundamental genotypic differences. Furthermore, the anther culturability of hybrid progeny is also influenced by parental genotypes. Typically, the anther culturability of hybrid rice follows the order: japonica hybrids > indica-japonica hybrids > indica hybrids [35], underscoring the significant impact of parental selection.

Sampling stage and position represent another crucial factor. The developmental stage of the young panicle directly affects the division state of pollen mother cells and their responsiveness to culture conditions. Research indicates that the late uninucleate stage of microspores is the most optimal for anther culture, whereas sampling either too early or too late can substantially reduce efficiency [36]. Therefore, accurately determining the developmental stage of young panicles is paramount. In practice, while precise assessment via bract removal and microscopic examination with 1% potassium iodide staining is possible, these methods are labor-intensive. Consequently, researchers often rely on external morphological cues, particularly the distance between the auricles of the flag leaf and the penultimate leaf (typically 5–10 cm, varying by variety) as a reliable indicator for sampling [37].

Pretreatment primarily functions to induce pollen grains into a developmental stage amenable to culture, promoting the formation of embryoids or callus. Appropriate pretreatment is beneficial for enhancing efficiency. Common methods include cold treatment, osmotic stress, and chemical treatment [38]. Cold treatment delays pollen degeneration, maintains a conducive physiological environment for pollen development, and can modulate endogenous hormone levels (e.g., increasing auxin, decreasing ethylene). As the cold tolerance of microspores varies by genotype, the optimal temperature and duration require adjustment, though 8–10 °C for 7–10 days is generally effective [39]. Osmotic stress, often induced by mannitol, regulates osmotic pressure within the anther, enhancing pollen tolerance and callus induction capacity. Chemical treatment, using agents like colchicine, alters the physico-chemical conditions within the anther to promote callus formation. Building upon cold pretreatment, subsequent treatment of isolated anthers and microspores with 60 g/L mannitol for 3 days or 10 mg/L colchicine for 3 days can further positively impact rice callus formation and green plant differentiation [40].

The growth environment of the donor plants is a significant factor. Suitable temperature, light, water, soil nutrition, and CO_2_ concentration ensure healthy plant growth and high-quality anther development, thereby improving culture efficiency. Studies show that temperature during rice growth directly influences pollen mother cell meiosis and microspore development. For instance, while pollen shedding typically occurs above 28 °C, temperatures rising to 33 °C severely impair pollen production, and temperatures reaching 39 °C lead to complete pollen inactivation, preventing germination on the stigma [41,42]. During microsporogenesis, the expression of tapetum-specific genes like YY1 and YY2 is significantly reduced under high-temperature stress, compromising pollen adhesion to the stigma and ultimately lowering germination rates [43]. Light intensity also affects the physiological state of anthers. Suitable light intensity enhances photosynthetic efficiency, promoting carbohydrate accumulation that provides essential nutrition for anther development. Huang et al. [44] reported significantly higher callus induction and green plant differentiation rates under natural light compared to shaded conditions. Water availability is another critical factor. Consistent and adequate water supply maintains normal physiological metabolism, ensuring proper pollen grain development. Zhang et al. [45] demonstrated that drought stress significantly inhibits rice plant growth, leading to marked reductions in plant height, spikelets per panicle, filled grains per panicle, seed set rate, yield per plant, and biomass per plant compared to well-watered controls, with these differences being highly significant.

The composition of the culture medium is vital for successful rice anther culture. Among its components, the carbon source, nitrogen source, and plant growth regulators (PGRs) are particularly crucial. The carbon source serves as the essential energy supply during culture, commonly provided as sucrose or maltose. Different carbon sources and their concentrations affect callus induction, differentiation, and green plant regeneration. Zhao et al. [46] found sucrose superior to maltose for callus induction in most japonica rice. Conversely, Li et al. [47] reported maltose more effective than sucrose in the induction medium for indica materials, yielding callus with stronger differentiation potential. Zhu et al. [48] advocated for a mixture of 3% sucrose and 3% maltose, suggesting it outperforms single carbon sources, potentially by meeting varying sugar metabolic demands across different developmental stages during callus induction [34]. The nitrogen source plays a key role in cell division and differentiation, supplied as inorganic nitrogen (nitrate or ammonium ions) and organic nitrogen (e.g., vitamins, amino acids) [49]. Zhu et al. [50] demonstrated that NH_4_^+^ concentration exerts a more profound influence on callus induction and differentiation than NO_3_^−^, SO_4_^2−^, or K^+^ concentrations. Low NH_4_^+^ significantly enhanced callus induction rate, while a slight increase drastically reduced it. Lu et al. [51] found that organic supplements like vitamin C, thiamine, inositol, and spermidine can promote callus formation, and suitable concentrations of hydroxyproline combined with proline can accelerate callus production. PGRs are chemicals that regulate plant growth and development at very low concentrations, categorized into natural plant hormones and synthetic compounds. They influence growth, organogenesis, flowering, fruiting, and stress resistance by modulating cell division, elongation, and differentiation. Based on function and mechanism, major classes of natural PGRs include Auxins, Cytokinins, Gibberellins (GAs), Abscisic Acid (ABA), and Ethylene. Commonly used synthetic PGRs in plant tissue culture, anther culture, and other breeding research include 2,4-dichlorophenoxyacetic acid (2,4-D), naphthaleneacetic acid (NAA), kinetin (KT), and 6-benzylaminopurine (6-BA) (Table 2). Lentini et al. [52], working with tropical japonica rice, found that combining 2,4-D (2 mg/L) with NAA (0.07 mg/L) was more effective than either auxin alone in promoting callus induction. KT is typically used in induction medium to improve callus quality, while 6-BA is commonly employed in differentiation medium to promote shoot regeneration and root formation from callus [53].

Rice anther culture is an integrated, multi-stage process requiring specific conditions at each phase. Zhang et al. [54], using indica-japonica F_1_ hybrids, investigated optimal temperatures for different stages. They recommended a pretreatment at 8 °C for 8 days, an induction culture temperature of 27–30 °C, and a differentiation culture temperature of 27 °C.

## 5. Genetic Mechanisms Underlying Rice Anther Culture Efficiency

During anther culture, factors such as young panicle pretreatment, culture medium, and incubation conditions are considered external influences on efficiency. These factors can be empirically optimized for different varieties to achieve the best possible outcomes. In contrast, the genotype of rice is regarded as the internal determinant and is the pivotal factor governing anther culture efficiency [55]. Genetic analyses have revealed the complex inheritance of this trait. Miah, using diallel cross analysis, demonstrated that callus induction rate (CIR) is controlled by genes with additive effects [56]. Subsequently, Quimio et al. found that both green plant differentiation rate (GPDR) and CIR share a similar genetic control pattern, primarily governed by additive gene action [57]. Conversely, Wu Chuanyin and Chen Ying reported that CIR is regulated not only by additive genes but also by non-additive gene effects [58]. Furthermore, He et al. observed no correlation between CIR and GPDR, indicating that these traits are controlled by distinct genetic factors [24]. Collectively, these studies establish that rice anther culture efficiency is a complex quantitative trait.

In 1998, the research team led by Zhu Lihuang at the Institute of Genetics, Chinese Academy of Sciences, performed quantitative trait locus (QTL) analysis for traits related to anther culture efficiency—including CIR, GPDR, albino plant differentiation rate (APDR), and green plant yield (GPY)—using a doubled haploid (DH) population of 132 lines. Their work led to the identification of five QTLs for CIR, two for GPDR, and one major QTL for APDR [24]. More recently, in 2021, Guo Tao’s team at South China Agricultural University employed a recombinant inbred line population comprising 192 lines and mapped eight QTLs associated with anther culture efficiency across three different environments. Among these, a major QTL for CIR, designated *qCIR9.1*, was consistently detected in all environments. Subsequent RNA-seq data analysis suggested that the candidate gene for *qCIR9.1* may encode a High Mobility Group (HMG) protein [59].

In DH populations derived from anthers of F_1_ hybrid plants, Mendelian genetics predicts a 1:1 segregation ratio for alleles at any locus from the male and female parents. However, numerous studies have reported widespread segregation distortion in such DH populations, where the observed allele frequencies deviate significantly from this expected ratio [60,61,62,63,64,65,66]. This segregation distortion is primarily attributed to the selection effect operating on microspores during in vitro anther culture. Microspores carrying alleles favorable for anther culture respond more readily to callus induction and green plant regeneration. This selective advantage leads to an overrepresentation of these favorable alleles in the resulting DH population. Consequently, when constructing genetic linkage maps from these DH populations, molecular markers linked to these loci exhibit segregation distortion [67,68].

Building on this concept, recent studies have leveraged segregation distortion analysis to identify loci associated with culturability. Dai et al. developed a DH population of 154 lines via anther culture of F_1_ plants from a cross between japonica restorer line SH26 and indica restorer line F38. Single-locus segregation distortion analysis pinpointed five additive loci linked to anther culture efficiency. Additionally, two-locus analysis revealed nine epistatic interaction locus pairs affecting efficiency [67]. In a separate study, Sun et al. generated a DH population of 232 lines through anther culture of young panicles from the hybrid japonica variety Shenyou 26 (SY26). Their analyses identified five additive effect loci and six pairs of epistatic loci related to anther culture efficiency via single-locus and two-locus segregation distortion analyses, respectively [68]. These findings provide a solid foundation for elucidating the genetic regulatory mechanisms controlling anther culture efficiency in rice. Nevertheless, the functional genes underlying the identified genetic loci remain largely unknown and warrant further in-depth investigation.

## 6. Current Challenges and Strategies in Rice Anther Culture

As a vital technique in haploid breeding, rice anther culture has played a significant role in genetic improvement and the development of new rice varieties. Nevertheless, several challenges persist that limit its broader application. The most critical constraint is the strong genotype dependence. Anther culture efficiency in indica rice is substantially lower than in japonica varieties, significantly restricting its utility in indica breeding programs. A common strategy to mitigate this limitation involves utilizing indica-japonica hybrids. This approach introduces high-efficiency alleles from japonica backgrounds into the progeny, thereby improving the genetic predisposition for successful in vitro anther culture and enhancing overall efficiency [33].

Another prevalent issue is the browning of anthers and callus. Anther browning occurs when anthers turn brown during culture due to cell death or the accumulation of phenolic compounds, ultimately impairing microspore differentiation and leading to failed callus induction. To address this, meticulous selection of anthers at the optimal developmental stage (typically the late uninucleate stage) and careful optimization of culture conditions—including temperature, light, and duration—are essential [69]. Callus browning, characterized by a brown discoloration resulting from phenolic oxidation or cell death, prevents normal differentiation and causes callus mortality. Countermeasures include not only optimizing the physical culture environment but also the timely subculture of newly formed callus to fresh medium, which helps reduce the buildup of phenolic compounds. Furthermore, supplementing the culture medium with additives such as glutathione (GSH), activated charcoal, or vitamin C can effectively alleviate browning [70].

A further challenge is the frequent occurrence of albino plantlets–chlorophyll-deficient plants that appear wholly or partially white or yellow and are incapable of photosynthesis. Research by He et al. [71] demonstrated that appropriately reducing the concentrations of inorganic salts and manganese in the induction medium, along with rationally adjusting the ratio of plant growth regulators like 2,4-D and KT, can not only improve the callus induction rate but also significantly reduce the frequency of albino plant regeneration.

In recent years, with the rapid development of multi-omics platforms such as genomics, transcriptomics, and proteomics, significant progress has been made in understanding the molecular mechanisms of rice anther culture. Recent research [72] has demonstrated that the *BBM-BAR1* gene module can efficiently reprogram the fate of microspores in rice anthers, substantially increasing the efficiency of asexual embryo formation. This process has been elucidated through techniques including transcriptomics and chromatin immunoprecipitation sequencing (ChIP-seq), revealing how the *BBM* gene regulates the expression of embryo-associated genes and thereby bypasses the need for traditional stress treatments. Looking forward, the continuous advancement of multi-omics platforms will enable a more comprehensive dissection of the complex networks governing gene expression, protein function, and metabolite dynamics during rice anther culture. These technologies will facilitate the precise optimization of culture conditions, reduce genotype dependence, and significantly improve culture efficiency.

## 7. Prospects

Rice anther culture technology, having evolved over nearly half a century, now constitutes an indispensable core component within haploid breeding systems, having contributed significantly to germplasm innovation and enhanced breeding efficiency. Nevertheless, its application within indica genetic backgrounds continues to face pronounced genotypic constraints. Persistent issues such as low culture efficiency and unstable regenerative capacity remain fundamentally unresolved. Research indicates that anther culture efficiency is inherently a complex quantitative trait governed by the synergistic action of multiple genes. The complete elucidation of its underlying genetic mechanisms and molecular regulatory networks remains an ongoing pursuit. Therefore, the systematic dissection of the molecular basis governing microspore dedifferentiation, callus formation, and plant regeneration during indica anther culture holds profound theoretical importance. Concurrently, it promises to provide a critical breakthrough for overcoming genotype dependency and achieving broader technical universality.

Recent years have witnessed rapid advances in molecular biology and genome editing, leading to groundbreaking progress in the development of in vivo haploid inducer lines in rice, thereby furnishing an alternative technical pathway for haploid induction. However, two major challenges impede the practical application of this technology: firstly, the induction efficiency of current inducer lines requires further enhancement to meet the demands of large-scale breeding programs; secondly, the self-pollinating nature of rice imposes constraints on efficient pollination by inducer lines and subsequent embryonic development, necessitating the exploration of compatible pollination systems and reproductive regulation strategies. The effective resolution of these issues will directly determine the adoption potential and application scope of inducer-mediated technology in breeding practice.

Looking forward, future efforts should be grounded in a deepened understanding of genetic mechanisms. Integrating multi-omics, gene editing, and smart breeding technologies will be crucial. A dual-track strategy, promoting the synergistic advancement of both anther culture and inducer-mediated techniques, should be pursued. This integrated approach will propel the rice haploid breeding technology system towards greater efficiency, wider applicability, and enhanced practicality, ultimately providing sustained technological support for rice genetic improvement and global food security.

## Figures and Tables

**Table 1:** Historical development of rice anther culture.

Period	Event	Material and Breeding Outcome	Impact	References
Foundation (1960s–1970s)	First successful report of haploid plant production via anther culture in Datura (1964).	–	Established the fundamental principle of microspore embryogenesis and the feasibility of haploid production in vitro.	[31]
	First successful report of anther culture in rice (1968).	–	Proved the concept for the most important food crop, initiating global research.	[32]
	Initiation of rice anther culture research and application in China (1970).	–	Marked the beginning of systematic efforts to apply the technology in a major rice-producing country.	–
Initial Application in Breeding (1970s–1980s)	Optimization of basic protocols (media, donor plant conditions) for japonica rice.	Danfeng 1 (Japonica, 1975)—The first rice variety developed via anther culture breeding in China.	Demonstrated the practical utility of anther culture for cultivar development, reducing breeding time significantly.	[33]
Scale-up and Diversification (1980s–1990s)	Improved induction and regeneration rates for specific japonica germplasms; protocol standardization.	Zhonghua Series (Zhonghua 8 to 14)—Developed by Chinese Academy of Agricultural Sciences. Longjing Series—Developed by Heilongjiang Academy. Huayu Series (Huayu 1, 2, 3, 13, 560, etc.)—Developed by Tianjin Institute.	Large-scale breeding success. Cumulative planting area (1975–1995): 0.91 million hectares. Popularized the technology and proved its economic impact.	[11]
Peak Adoption and Impact (1990s)	Widespread adoption and integration into breeding programs of major institutes for japonica rice.	Over 40 approved varieties by 1998.	Planting area (1996–1998 alone): 0.95 million hectares, exceeding the total of the previous 21 years. Represented the peak impact of the technology on production.	[11]
Challenges and Mechanistic Research (2000s–Present)	Recognition of strong genotype dependence; particular difficulty in indica rice. Limited application in modern breeding pipelines.	Limited number of new varieties released recently; technology primarily maintained in few specialized institutions.	Highlighted the major limitation restricting broader application. Shifted research focus towards understanding genetic and molecular bases of culturability.	(This review)

**Table 2:** Plant hormones and formulations.

Plant Hormones	Concentration	Mother Liquor
2,4-Dichlorophenoxyacetic acid (2,4-D)	2 mg/L	0.2 g dissolved in 10 mL ethanol
α-Naphthaleneacetic acid (NAA)	1 mg/L	0.2 g dissolved in 10 mL ethanol
Kinetin (KT)	1 mg/L	0.2 g dissolved in 10 mL DMSO
6-Benzylaminopurine (6-BA)	2 mg/L	0.2 g dissolved in 10 mL DMSO
Paclobutrazol (MET)	2.5 mg/L	0.2 g dissolved in 10 mL ethanol

## Data Availability

No new data were created or analyzed in this study.
